# Leigh syndrome in an infant: autopsy and histopathology findings

**DOI:** 10.4322/acr.2021.334

**Published:** 2021-11-12

**Authors:** Arushi Gahlot Saini, Debjyoti Chatterjee, Chandana Bhagwat, Sameer Vyas, Savita Verma Attri

**Affiliations:** 1 Postgraduate Institute of Medical Education and Research, Department of Pediatrics, Chandigarh, India; 2 Postgraduate Institute of Medical Education and Research, Department of Histopathology, Chandigarh, India; 3 Postgraduate Institute of Medical Education and Research, Department of Radiodiagnosis and Imaging, Chandigarh, India

**Keywords:** Basal Ganglia, Brain Damage, Chronic, Leigh Disease, Mitochondrial Diseases

## Abstract

Leigh syndrome is an inherited neurodegenerative disorder of infancy that typically manifests between 3 and 12 months of age. The common neurological manifestations are developmental delay or regression, progressive cognitive decline, dystonia, ataxia, brainstem dysfunction, epileptic seizures, and respiratory dysfunction. Although the disorder is clinically and genetically heterogeneous, the histopathological and radiological features characteristically show focal and bilaterally symmetrical, necrotic lesions in the basal ganglia and brainstem. The syndrome has a characteristic histopathological signature that helps in clinching the diagnosis. We discuss these unique findings on autopsy and radiology in a young infant who succumbed to a subacute, progressive neurological illness suggestive of Leigh syndrome. Our case highlights that Leigh syndrome should be considered in the differential diagnosis of infantile-onset, subacute neuroregression with dystonia and seizures, a high anion gap metabolic acidosis, normal ketones, elevated lactates in blood, brain, and urine, and bilateral basal ganglia involvement.

## INTRODUCTION

Leigh syndrome is an inherited neurometabolic and neurodegenerative disorder of infancy that typically manifests between 3 and 12 months of age. Dr. Dennis Archibald Leigh^1^ first described the disorder as a ‘subacute necrotizing encephalomyelopathy’ in 1951 based on the autopsy findings in a 7-month-old boy. The typical clinical features are developmental delay or regression, progressive cognitive decline, dystonia, ataxia, brainstem dysfunction, epileptic seizures, and respiratory dysfunction.^2^ Additional features include weakness, fatigue, hypotonia, tremor, poor sucking and feeding, ptosis, nystagmus, abnormal ocular findings, and failure to thrive. Although the disorder is clinically and genetically heterogeneous, the histopathological and radiological features characteristically show focal and bilaterally symmetrical, necrotic lesions in the basal ganglia and brainstem. The term ‘Leigh-like syndrome’ is often used when partial or atypical symptoms, laboratory findings, or radiologic features are present, but the overall clinical picture indicates Leigh syndrome. The syndrome has a characteristic histopathological signature which may help in clinching the diagnosis. More than 75 genes and different inheritance patterns have been associated with Leigh syndrome. The disorder is most commonly inherited in autosomal-recessive pattern associated with pathogenic variations in the nuclear-encoded mitochondrial genes. Less commonly, the disorder may have a maternal inheritance pattern associated with pathogenic variations in the genes in the mitochondrial DNA. In today’s era of genomics, the histopathological findings of this distinct disorder are rarely described.^3^^,^^4^ We discuss these unique findings on autopsy and radiology in a young infant who succumbed to a subacute, progressive neurological illness suggestive of Leigh syndrome.

## CASE REPORT

A 7-month-old boy presented with progressive deterioration in sensorium, seizures, intermittent dystonia, and neuroregression for three weeks, following a trivial fall from the bed. He gradually lost motor milestones, speech, and eye contact. He stopped recognizing his parents, became lethargic, less interactive, and did not demand feeds. He also developed intermittent, generalized abnormal twisting of the limbs, which increased during awake state or stimulation while reduced during sleep. He had four episodes of intermittent, generalized tonic stiffening with up rolling of eyeballs, lasting 2-3 minutes each and associated with postictal drowsiness. There was no history of fever, systemic illness, abnormal body odor, rash, or hyperpigmentation. He was born to non-consanguineous parents with two older female siblings who were alive and well. He was born full term and the perinatal period was normal. On examination, his weight was 7 kg (-1 to -2 z) and head circumference 41.8 cm (-2 to -3 z). He had a low Glasgow Coma Score of 6 (eye opening 1, verbal response 2, and motor response 3), pallor, mild hepatomegaly (span 7 cm), axial hypotonia, intermittent generalized dystonia, brisk deep tendon reflexes, flexor plantar response, normal fundi, and absence of ophthalmoplegia or facial nerve involvement. A clinical diagnosis of subacute, afebrile encephalopathy was considered. He was administered empiric antimicrobials, intravenous fluids containing glucose, and supportive care.

**Figure 1:**
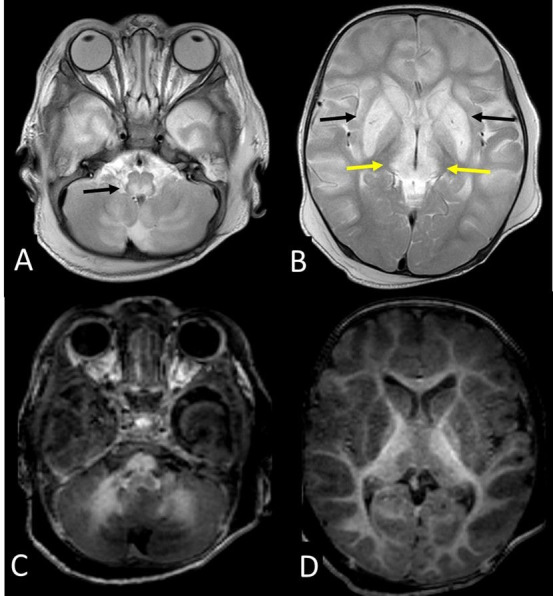
Axial T2-weighted (**A** and **B**) and T1-weighted (**C** and **D**) MR images show bilateral symmetric signal changes which were hyperintense on T2 and hypointense on T1-weighted images, involving brainstem dorsal tracts (black arrow, **A**), basal ganglia (black arrows, **B**), medial thalami (yellow arrows, **B**), with diffuse cerebral gyral swelling (more marked in fronto-temporal lobes) and cystic changes in bilateral putamina.

**Figure 2:**
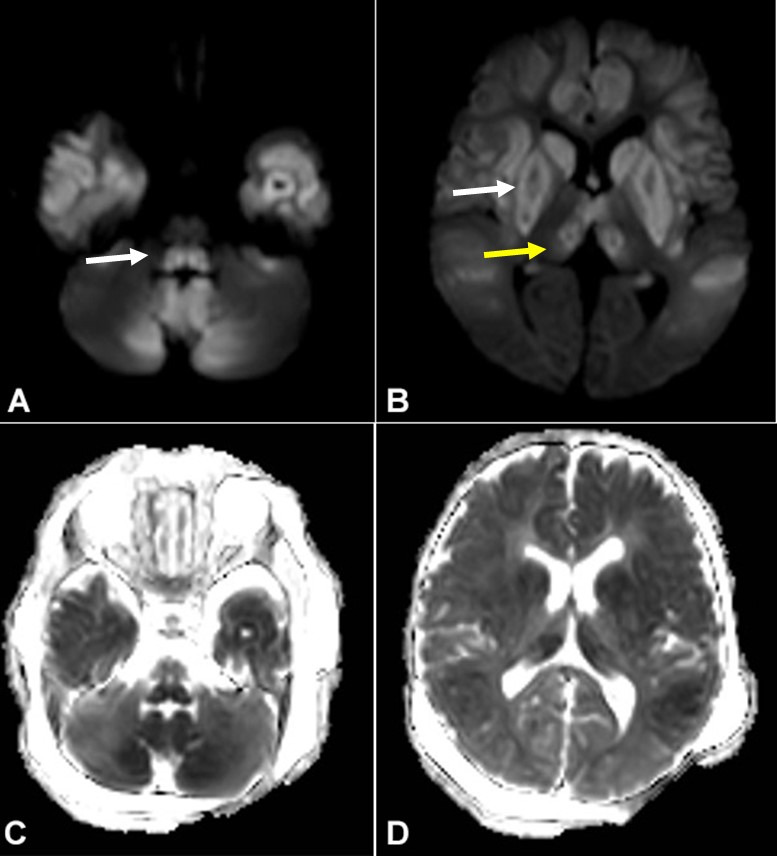
Axial diffusion-weighted imaging (DWI) (**A** and **B**. b = 1000 s/mm^2^) and apparent diffusion coefficient (ADC) maps (**C** and **D**) show bilateral diffusion restriction involving brainstem dorsal tracts (white arrow, **A**), basal ganglia (white arrow, **B**), medial thalami (yellow arrow, **B**), cerebellum and diffuse cerebral gyral swelling (more marked in fronto-temporal regions).

**Figure 3:**
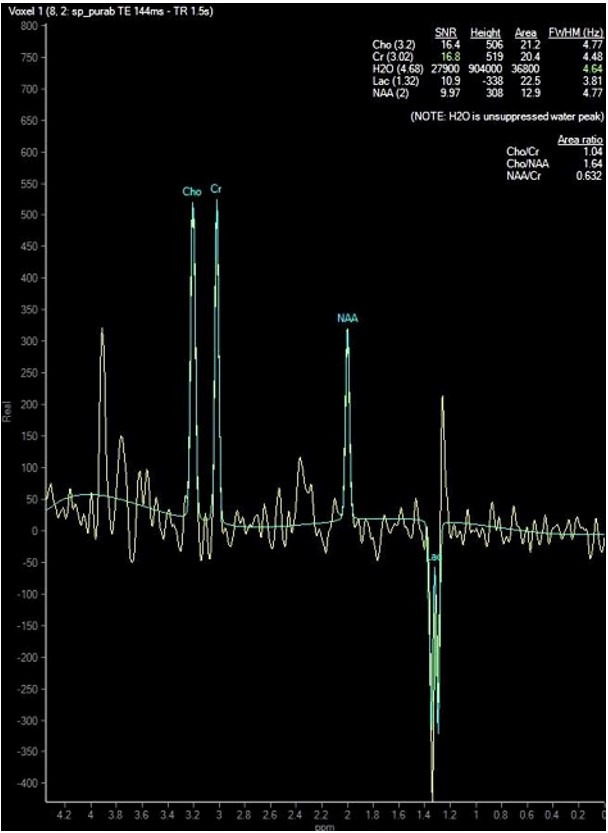
MR spectroscopy (TE=144ms) at basal ganglia (putamen) showing lactate peak (double peak at 1.3 ppm which is inverted) and decreased NAA peak.

Laboratory studies showed anemia (hemoglobin 9,4 g/dL) with low mean corpuscular volume (71 fL, reference range [RR] 80-100 fL) and transaminitis (aspartate aminotransferase 1228 IU/L, RR 5-40 IU/L, and alanine aminotransferase 784 IU/L, RR 5-40 IU/L). The platelets, total leucocyte count, prothrombin index, serum electrolytes, and renal function tests were normal. Arterial blood gas analysis showed high anion gap metabolic acidosis (pH 7.26, bicarbonate 13.4, and anion gap 20). His blood lactate level at admission was 9.9 mmol/L and remained between 8 and 18 mmol/L (normal range 1-2 mmol/L) throughout the course. Blood ketones and sugar were normal. Magnetic resonance imaging (MRI) of the brain showed bilateral symmetric lesions which were hyperintense on T2 and hypointense on T1-weighted images (Figure 1) with diffusion restriction (Figure 2) in cerebral hemisphere gyri, deep grey structures, and dorsal brainstem with lactate peak (Figure 3).

Based on combined clinical, laboratory, and imaging findings, a neurometabolic disorder such as Leigh syndrome (energy deficiency disorder), or organic acidemia (intoxication disorder), or biotin-thiamine-responsive basal ganglia disease were considered. He was started on multivitamin and cofactor supplements, including high-dose biotin (10 mg/kg/d) and thiamine (300 mg/day). Hyperglycemia developed later during the hospital course and insulin infusion was started to maintain normoglycemia. For persistent metabolic acidosis, he was treated with intravenous sodium bicarbonate. Tandem mass spectroscopy revealed normal amino acids and acylcarnitine profiles. Gas chromatography showed significant lactic acid excretion in the urine, supporting a mitochondrial respiratory chain defect. Genetic studies could not be done due to the non-availability of these tests at our center. His condition worsened with hypotensive shock, transaminitis, persisting lactic acidosis, and multi-organ dysfunction syndrome. He succumbed to his illness on day four of hospitalization, and an autopsy was conducted with parental consent.

## AUTOPSY FINDINGS

**Figure 4:**
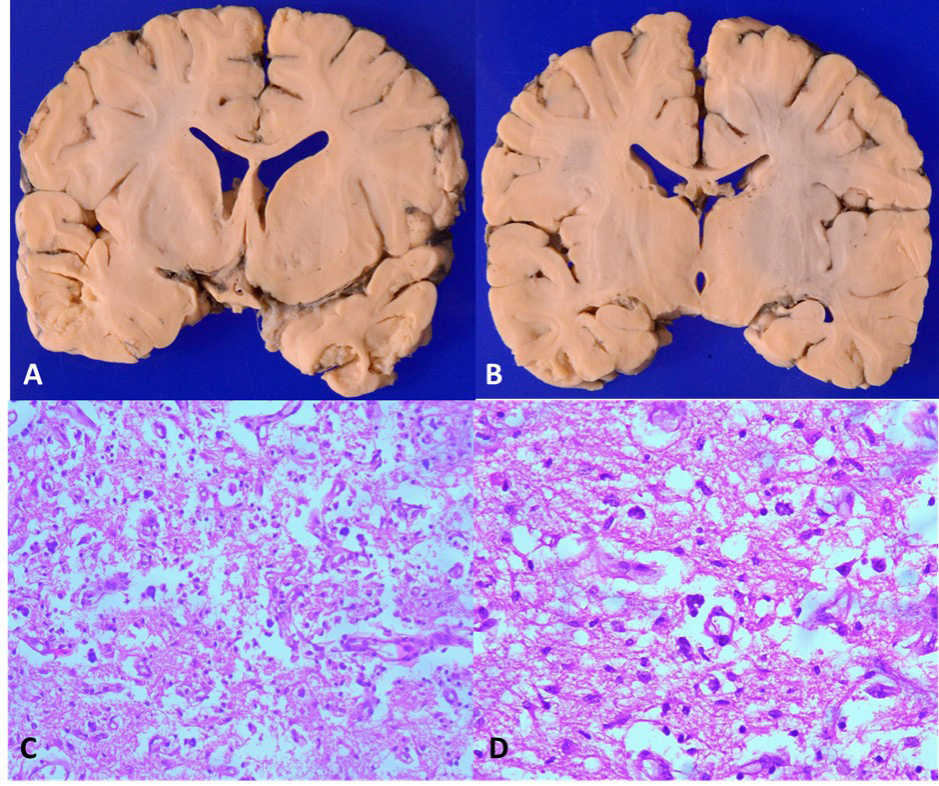
Coronal slices of brain at the level of basal ganglia in **A** and thalamus in **B** shows multiple cystic spaces with granularity involving bilateral caudate nucleus, putamen, globus pallidum, thalamus and temporal cortex; **C –** Section from putamen shows neuropil vacuolization, gliosis and vascular proliferation (H&E, x100); **D –** Multiple foci of microcalcification were also noted (H&E, x200).

At autopsy, the brain weighed 940 g (normal 700-750 g). There was presence of cerebral edema which involved diffusely the cortex as well as deep grey matter. Externally, the leptomeninges were clear without exudates. Coronal sectioning of the brain revealed mild softening and granularity areas involving the bilateral caudate nucleus, putamen, globus pallidum, and thalamus with focal areas of cystic changes (Figure 4A, B). Microscopic examination of the sections taken from the mammillary bodies did not reveal any significant pathology. The periaqueductal grey matter appeared discolored. The temporal cortex showed mild granularity. Microscopic examination of the brain revealed primary pathology involving the central grey matter. There was extensive neuropil vacuolization with rarefaction, marked astrogliosis, vascular proliferation, and focal calcification with intact neurons (Figure 4) in bilateral globus pallidum, putamen, and periaqueductal grey matter. There were few foci of macrophage collection in the putamen. These were small foci, centered around the vessels and showed presence of dystrophic calcification in the adjacent areas.

**Figure 5:**
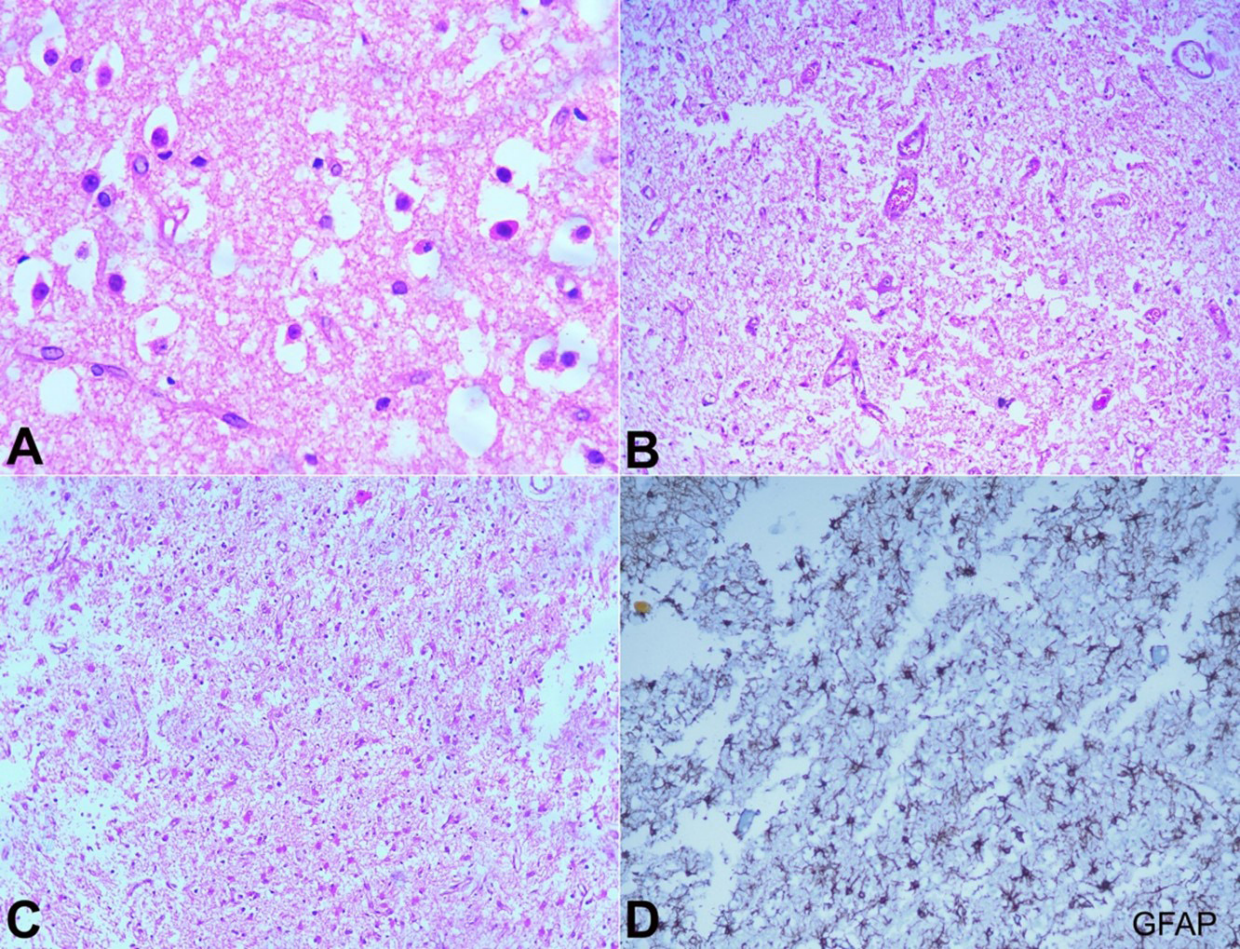
**A –** Section from the putamen shows preserved neurons, however, they show pyknotic nuclei and cytoplasmic hypereosinophilia (H&E, x400); **B –** Section from midbrain shows marked loss of neurons with an occasional preserved neuron (H&E, x200); **C –** Midbrain shows extensive reactive gliosis (H&E, x100), as highlighted in **D –** by GFAP stain (x200).

Neurons showed pyknotic nuclei and hypereosinophilic cytoplasm. Caudate nuclei and thalami also showed similar changes, but to a lesser extent (Figure 5). The internal capsule was relatively spared. The temporal and frontal cortices showed neuropil vacuolization and pyknotic neurons.

Lungs were subcrepitant. The right upper lobe showed the presence of hemorrhage. Microscopic examination revealed occasional fibrin thrombi in pulmonary artery branches. Sections from the right upper lobe showed diffuse alveolar hemorrhage and early bronchopneumonia. Kidneys showed medullary congestion. Microscopic examination revealed multiple thrombi within glomerular capillaries. Microscopic examination of the liver revealed maintained lobular architecture with centrical hemorrhagic necrosis, probably due to hypotensive shock. Focal macro- and microvesicular steatosis were also observed. Other organs did not show any significant pathology. Electron microscopy from the skeletal muscle did not reveal any mitochondrial structural abnormality. Biochemical analysis of muscle was not performed. Muscle histology did not reveal any significant pathology. There were no subsarcolemmal deposition or any ragged-red fibers on modified Gomori's stain. No COX deficient fibers were seen. However, respiratory chain enzyme analysis or genetic studies could not be performed postmortem as these facilities were not available at our centre. Overall, the autopsy diagnosis was necrotizing lesions involving bilateral basal ganglia, thalamus, and midbrain consistent with Leigh syndrome, with early bronchopneumonia, pulmonary hemorrhage, and disseminated intravascular coagulation.

## DISCUSSION

Our case presented with subacute neuroregression with progressive encephalopathy, dystonia, seizures, and respiratory failure that were possibly triggered by trivial head trauma or an unnoticed infection. The biochemical parameters showed a high anion gap metabolic acidosis, persistent lactic acidosis, normal ketones, which was highly suggestive of an inborn error of metabolism involving the energy pathways such as the mitochondria. The radiology showed characteristic bilateral, symmetrical basal ganglia and brainstem involvement, which are the mitochondria-rich parts of the brain.

The pathophysiological basis in Leigh syndrome is the disruption of ATP synthesis, which may be due to interruption of any of the three steps of energy generation after glycolysis (a) oxidation of pyruvate into acetyl Co-A via pyruvate dehydrogenase complex (PDHc) (b) citric acid cycle (Kreb’s cycle) (c) electron transport chain (oxidative phosphorylation) involving the respiratory chain complexes I-V. The common causes of Leigh syndrome are listed in Table 1.

**Table 1:** Causes of Leigh syndrome^12^

**Mitochondrial**
· Defects in pyruvate dehydrogenase complex enzymes
· Defects in citric acid cycle
· Defects in electron transport chain (oxidative phosphorylation) including coenzyme Q
o Defects in nuclear DNA
o Defects in mitochondrial DNA
**Treatable causes of Leigh syndrome**
· Defects in Coenzyme Q
· Pyruvate dehydrogenase enzyme
· Biotin-thiamine responsive basal ganglia disease
· Biotinidase deficiency (additionally catalyzes posttranslational modification of histones by biotinylation)
· Succinyl-CoA ligase deficiency with methyl malonic aciduria (*SUCLA2* gene associated)
**Non-mitochondrial causes of Leigh syndrome**
· 3-methyl-glutaconic aciduria

Amongst the respiratory chain disorders, complex I is the largest complex and is most commonly affected. Biochemical evaluation and specific metabolic profile suggest impairment in energy production in the mitochondria, as seen in the index case with persistent hyperlactatemia and metabolic acidosis.^6^ Blood pyruvate levels could not be tested as these are not available at our center. However, the characteristic radiological and histopathological picture confirm the diagnosis of Leigh syndrome in this patient. Genetic testing could not be done due to financial constraints. The clinical features and investigations point towards a nuclear DNA-mediated Leigh syndrome. A recent meta-analysis of 385 patients showed that patients with pathogenic variations in nuclear DNA are significantly younger than those with mitochondrial DNA variants and have more involvement of the medulla and the cerebellum.^2^ Biotin-thiamine responsive basal ganglia disease is a close differential diagnosis, which should always be considered a treatable cause of Leigh-like presentation.^7^

The outcome in Leigh syndrome is generally poor, with a median age at death of 2.4 years. The cause of death is most commonly respiratory failure and infections. Persistently increased lactate predisposes to further infection by altering immune cell function in its local environment.^8^ Acute respiratory failure is a frequent feature of Leigh seen in 64-72% of cases.^9^ The early features are irregular breathing, recurrent apnea, deep sighing respiration, unexplained hyperventilation, and hiccups with lethargy, which may manifest weeks or months before a definitive respiratory failure. However, the latter may also occur without a prodrome and is due to the involvement of the brainstem with or without the involvement of respiratory muscle weakness. The mild hepatomegaly may be a part of the underlying illness in this patient. However, the preterminal marked elevation of liver enzymes (aspartate aminotransferase 1228 IU/L, RR 5-40 IU/L and alanine aminotransferase 784 IU/L, RR5-40 IU/L) was perhaps secondary to a combined effect of shock, hypoxia, and systemic dysfunction with a possible underlying predisposition due to the mitochondrial disease. Liver failure has been seen as a late complication of Leigh disease.^10^

A unique feature of this case is the short clinical history (3 weeks of progressive neurodegeneration) and rapid progression to death only 4 days after presentation. Besides the necrotization and irreversible damage in the central cardiorespiratory control areas in the brainstem, a rapid failure of the energy systems in the brain, muscles, and other organs can result in rapid coma and death. Combined together, this can lead to respiratory muscle paralysis contributing to early death. Sudden and unexpected death in children and adults with Leigh syndrome can be caused by various and complex cardiac, neurological, and/or metabolic mechanisms including acute metabolic acidosis, cerebral necrosis/edema, epilepsy, cardiac arrhythmia, respiratory failure, aspiration of gastric contents, asphyxia, and hypertrophic cardiomyopathy.^4^^,^^5^

Several factors determine the outcome in Leigh syndrome. Increased cerebrospinal fluid lactate levels are markers of poor outcome, although one-fourth of cases may have normal lactate in the blood or cerebrospinal fluid throughout the disease course.^2^ Other markers of poor outcome include a lactate peak, lesions in the regions besides the basal ganglia, especially the brainstem, onset of disease in early infancy, failure to thrive, and intensive care requirement in care.^11^ Several of these predictors were also noted in the index patient and portended a poor prognosis.

## CONCLUSION

Leigh syndrome should be considered in the differential diagnosis in infantile-onset, subacute neuroregression with dystonia and seizures, a high anion gap metabolic acidosis, normal ketones, elevated lactates in blood, brain, and urine, and bilateral basal ganglia involvement.

## References

[1] Leigh D (1951). Subacute necrotizing encephalomyelopathy in an infant. J Neurol Neurosurg Psychiatry.

[2] Chang X, Wu Y, Zhou J, Meng H, Zhang W, Guo J (2020). A meta-analysis and systematic review of Leigh syndrome: clinical manifestations, respiratory chain enzyme complex deficiency, and gene mutations. Medicine.

[3] Lake NJ, Bird MJ, Isohanni P, Paetau A (2015). Leigh syndrome: neuropathology and pathogenesis. J Neuropathol Exp Neurol.

[4] Ventura F, Rocca G, Gentile R, De Stefano F (2012). Sudden death in Leigh syndrome: an autopsy case. Am J Forensic Med Pathol..

[5] Wick R, Scott G, Byard RW (2007). Mechanisms of unexpected death and autopsy findings in Leigh syndrome (subacute necrotising encephalomyelopathy). J Forensic Leg Med.

[6] Schubert Baldo M, Vilarinho L (2020). Molecular basis of Leigh syndrome: a current look. Orphanet J Rare Dis.

[7] Saini A, Sharma S (2021). Biotin-thiamine-responsive basal ganglia disease in children: a treatable neurometabolic disorder. Ann Indian Acad Neurol.

[8] Nolt B, Tu F, Wang X (2018). Lactate and Immunosuppression in Sepsis. Shock.

[9] Mak SC, Chi CS, Tsai CR (1998). Mitochondrial DNA 8993 T > C mutation presenting as juvenile Leigh syndrome with respiratory failure. J Child Neurol.

[10] Chinnery PF, DiMauro S (2005). Mitochondrial hepatopathies. J Hepatol.

[11] Sofou K, De Coo IF, Isohanni P (2014). A multicenter study on Leigh syndrome: disease course and predictors of survival. Orphanet J Rare Dis.

[12] Wortmann S, Rodenburg RJ, Huizing M (2006). Association of 3-methylglutaconic aciduria with sensori-neural deafness, encephalopathy, and Leigh-like syndrome (MEGDEL association) in four patients with a disorder of the oxidative phosphorylation. Mol Genet Metab.

